# Verbal fluency in breast cancer patients treated with chemotherapy

**DOI:** 10.1007/s12282-016-0713-4

**Published:** 2016-07-19

**Authors:** Paulina Andryszak, Monika Wiłkość, Bogdan Żurawski, Paweł Izdebski

**Affiliations:** 1 Institute of Psychology, Kazimierz Wielki University, Staffa 1, 85-867 Bydgoszcz, Poland; 2 Department of Psychiatry, Collegium Medicum in Bydgoszcz, Nicolaus Copernicus University in Torun, Bydgoszcz, Poland; 3 The Franciszek Lukaszczyk Oncology Center in Bydgoszcz, Bydgoszcz, Poland

**Keywords:** Verbal fluency, Cognition, Breast cancer, Chemotherapy

## Abstract

**Background:**

Cognitive decline caused by chemotherapy used in the treatment of malignant diseases was reported in several studies. ICCTF recommends the diagnosis of cognitive function in patient treated with chemotherapy. One of the suggested method is Verbal Fluency Test (VFT).

**Methods:**

Study was carried out on a group of 30 women with early breast cancer treated with adjuvant chemotherapy and 29 healthy controls. The patients underwent neuropsychological assessment using VFT at three time points: T1: before chemotherapy, T2: mid-chemotherapy and T3: post-chemotherapy. The examination in healthy controls was conducted at the same time intervals.

**Results:**

In phonetic fluency task patients produced more words at T2 compared to T1 (*Z* = 2.02; *p* < 0.05) and at T3 compared to T1, both patients (*Z* = 2.36; *p* < 0.05) and controls (*Z* = 2.57; *p* < 0.01). The patients scored lower than controls (*Z* = −2.04; *p* < 0.05) as well as on average cluster size in the same task (*Z* = −2.38; *p* < 0.05) at T3, while they scored higher on the number of phonetic switches at T2 compared to T1 (*Z* = 2.62; *p* < 0.01) and at T3 compared to T1 (*Z* = 2.50; *p* < 0.01). In semantic task controls produced more words at T3 than at T1 (*Z* = 2.62; *p* < 0.01) and at T3 compared to T2 (*Z* = 2.89; *p* < 0.01) and semantic clusters at T3 compared to T2 (*Z* = 2.43; *p* < 0.05). In patients, number of clusters was smaller at T3 compared to T2 (*Z* = −2.85; *p* < 0.05), while number of semantic switches was higher at T3 than at T2 (*Z* = 3.05; *p* < 0.01). Patients scored also lower than controls on number of semantic switches at T2 (*Z* = −2.05; *p* < 0.05).

**Conclusions:**

Chemotherapy does not decrease verbal fluency, but it has a negative impact on semantic memory.

## Introduction

In the last two decades, much attention was paid to the negative cognitive changes occurring as a result of chemotherapy used in the treatment of malignant diseases [1]. The International Cancer and Cognition Task Force (ICCTF) recommends the evaluation of cognitive performance of chemotherapy patients. The suggested methods include verbal fluency tests [1, 2]. The indicator of the level of performance is the number of produced words matching the criterion (usually words with a specific initial letter or belonging to a given category) and the number of errors (perseverations, neologisms, words unrelated to the criterion). Moreover, using quantitative analysis it is possible to evaluate the sizes of produced word clusters and switches between the groups [3–5]. The number of listed terms and the content of clusters reveal the structure of semantic memory, while switches are associated with executive functions [4, 5]. The fluency of word production is thus dependent not only on the verbal functions but also on other cognitive processes, including psychomotor speed, attention, or memory (semantic, episodic, and working memory) [6, 7], as well as the efficiency of executive functions [8]. It has also been supported by the results of neuroimaging studies, which revealed the activation of various areas of the brain, including the frontal and temporal lobes of the left hemisphere, during the performance of tasks testing verbal fluency [3, 6]. During the production of words belonging to various semantic classes or beginning with a given sound (associative aspect of fluency) the activation of temporal areas increases, while the activity of prefrontal areas (anterior cingulate gyrus) increases during transitioning between groups (switches, executive aspect of speech) [9].

The results of the most recent meta-analysis of studies carried out on patients treated with chemotherapy [2] showed an impairment of cognitive functions involved in the performance of tasks which measure verbal fluency, i.e. the capacity and selectivity of attention as well as immediate and delayed verbal memory compared to healthy people. Researchers also noted deficits in executive functions (EF) among BC survivors, such as working memory, cognitive flexibility or multitasking [10]. Moreover, the results of neuroimaging studies indicate that chemotherapy is associated with functional and structural changes in prefrontal cortex, which is a crucial neural region for EF [10, 11]. It has been also shown, that the activation of the left frontal area [12] and prefrontal lobe [10, 13] while performing tasks testing executive functions is decreased in women with breast cancer after chemotherapy and that the density of the gray matter in the left middle and superior frontal gyri in women with breast cancer was lower 1 month post-chemotherapy [10, 12]. Several mechanisms for chemotherapy-induced neurocognitive dysfunctions and abnormalities in brain structure and function were proposed, however, mechanisms that underlie them remain still unclear [14]. Some of the studies showed compromised cognitive function and brain abnormalities before the introduction of adjuvant chemotherapy treatment, e.g., executive network inefficiency, suggesting different risk factors, such as psychological distress or fatigue [15–19] Recently, the results of preclinical studies provided evidence that the combination of doxorubicin and cyclophosphamide negatively affected hippocampal neurogenesis [20] and impact synaptic plasticity and caused aging of molecules [21].

Only a few studies involved the evaluation of verbal fluency in patients treated with chemotherapy for cancer [22–29], without yielding unambiguous results. The majority of papers [24, 25, 27, 28] failed to demonstrate changes in verbal fluency according to phonetic criterion (mainly the letters F, A, and S) following chemotherapy. Two of the studies revealed a decreased phonetic verbal fluency in women with breast cancer immediately after and 3 months after completing adjuvant chemotherapy [26] and a decrease in phonetic and semantic fluency before the end of neoadjuvant chemotherapy [23]. In the latest research it was shown that the semantic fluency (regarding names of animals) is significantly lower in women treated with adjuvant chemotherapy than in healthy women [30].

The aim of the study was to analyze the influence of anthracycline-based adjuvant chemotherapy (4 cycles of Doxorubicin—Cyclophosphamide: AC chemotherapy) used in the treatment of breast cancer on verbal fluency evaluated with a phonetic and semantic fluency test.

## Patients and methods

### Participants

The study was carried out on a group of women with early breast cancer (BC group, *n* = 30) scheduled for adjuvant chemotherapy, and a group of healthy controls (HC group, *n* = 29). Women with breast cancer were recruited at the Chemotherapy Outpatient Clinic of the Oncology Center in Bydgoszcz. The study enrolled women with invasive breast cancer (Stages I-IIIA), mean age of 52.5 years (SD = 8.06) and mean education of 13.9 years (SD = 3.38), who were scheduled for adjuvant chemotherapy. All BC patients received four doses of AC chemotherapy: Doxorubicin 60 mg/m2 + Cyclophosphamide 600 mg/m^2^ every 21 days. Women in BC group were in good general conditions (performance status 1 on the Zubrod scale: restricted in physically strenuous activity and able to carry out work of a light or sedentary nature; completely ambulatory). The healthy controls were matched to the BC group women for age (mean age of 51.8, SD = 8.13 years) and education (mean education of 14.3 years, SD = 3.42). Participants with history of anti-neoplastic therapy, signs of damage to the CNS, mental disorders, addiction to psychoactive substances, concomitant diseases, or taking medication hindering contact with the surroundings were excluded from the study. Sociodemographic and clinical data of BC group are presented in Table 1. The study was approved by the Bioethical Committee of the Collegium Medicum in Bydgoszcz. Informed consent was obtained from all individual participant included in the study.

**Table 1:** Sociodemographic and clinical data of BC group compiled

Number of participants	30
Age (mean ± SD)	52.5 ± 8.06
Education (years ± mean)	13.9 ± 3.38
Breast cancer (*N*; %)	
Right	13; 43
Left	17; 57
Stage according to TNM (*N*; %)	
IA	16; 53 $\frac{1}{3}$
IIA	10; 33 $\frac{1}{3}$
IIB	3; 10
IIIA	1; 3 $\frac{1}{3}$
Elston–Ellis histologic malignancy grade (*N*; %)	
G1—low	1; 3
G2—medium	21; 70
G3—high	8; 27
Receptor expression (*N*; %)	
Estrogen receptor positive (ER+)	24; 80
Progesterone receptor positive (PR+)	17; 57
Overexpression of HER2/neu receptor (HER2+)	11; 37
Triple negative: ER-, PR-, HER-	7; 23
Hormonal status (*N*; %)	
Pre-menopausal	13; 43
Post-menopausal	17; 57
Surgical treatment method (*N*; %)	
Quadrantectomy	18; 60
Mastectomy	12; 40
Number of surgeries (*N*; %)	
1	23; 77
2	6; 20
3	1; 3
Time between surgery and first chemothearpy (days ± SD)	42 ± 10.46
Other somatic diseases	11; 37

### Study design

The study was longitudinal. The examination was carried out in the BC group at 3 time points: T1—before chemotherapy, T2—mid-chemotherapy (just before 3rd treatment cycle), and T3—post-chemotherapy (up to 30 days after the last chemotherapy cycle ended). The examination in the HC group was conducted at the intervals matching those of the BC group.

### Neuropsychological assessment

A verbal fluency test was one of the tools used to measure cognitive functions in the context of a larger research project. The study used two types of tasks to assess the verbal fluency over 60 s according to a preset phonetic or semantic criterion (respectively, listing as many words as possible which begin with a given letter, excluding proper names, or as many words as possible belonging to a specific category). The letters K, P, S were used for the study, as they are recommended as Polish equivalents of the English F, A, S, as well as the names of animals, fruits and vegetables [3, 4]. Because of the lack of data on the frequencies of words beginning with any given sound, the exact same set of letters and categories was used in all measurements on purpose. The word count was the number of spoken words minus repetitions and words which did not comply to the instructions. Two or more subsequent words related semantically or phonetically, were considered a cluster. Switches were related to a change in the answer selection strategy ending the current cluster. The mean cluster size (the sum of words in each cluster minus 1 divided by the number of clusters) was also calculated. To demonstrate the significant properties, it was decided to adopt the following measures of performance: the sum of produced word meeting the phonetic and semantic criterion (letters K, P, S and animals, fruit, and vegetables) and, respectively, the sums of clusters, average-sized clusters, switches, and errors.

### Statistical analysis

The statistical analysis of the data was carried out using STATISTICA 12 software. Most variables deviated from normal distribution. Therefore, we used: the Friedman’s ANOVA test for multiple samples, and the Wilcoxon signed-rank test for two. Also, the Mann–Whitney *U* test was applied to determine significant differences between the experimental and control groups.

## Results

### Phonetic fluency

It was found that in the task measuring phonetic fluency patients from the BC group produced significantly more words at T2 compared to T1 (*Z* = 2.02; *p* < 0.05) and at T3 compared to T1, both in the BC group (*Z* = 2.36; *p* < 0.05) and the HC group (*Z* = 2.37; *p* < 0.01) (Table 2).

**Table 2:** Differences between the results before (T1), during (T2), and after chemotherapy (T3) in patients with cancer (BC) and healthy controls (HC), evaluated with the ANOVA Friedman test (*N* = 30, df = 2) and the Wilcoxon’s signed-rank test

		BC				HC			
		T1–T3	T1&T2	T1&T3	T2&T3	T1–T3	T1&T2	T1&T3	T2&T3
		*χ* ^2^	*Z*	*Z*	*Z*	*χ* ^*2*^	*Z*	*Z*	*Z*
Phonetic fluency	Word count	5.74	2.02*	2.36*	0.44	6.11*	1.03	2.37*	1.28
	Error count	6.42*	1.46	0.02	1.91	1.75	0.31	0.44	0.75
	Cluster count	0.34	0.64	0.73	0.30	6.02*	0.79	2.22*	1.27
	Mean cluster size	1.73	0.56	0.80	2.01*	0.13	0.10	0.22	0.05
	Switch count	10.24**	2.62**	2.50**	0.38	0.49	0.58	0.95	0.04
Semantic fluency	Word count	0.68	0.64	0.53	0.22	6.07*	0.26	2.62**	2.89**
	Error count	2.60	0.41	1.50	1.69	2.24	1.49	0.94	0.09
	Cluster count	2.80	0.14	1.29	1.41	4.17	1.47	0.75	2.43*
	Mean cluster size	8.59**	1.53	0.95	2.85**	0.49	1.07	0.88	0.16
	Switch count	6.13*	1.17	1.42	3.05**	0.28	0.63	0.31	0.83

*T1* examination before, *T2* during, *T3* after chemotherapy, *χ* ^*2*^ empirical value of the ANOVA Friedman test, *Z* empirical value of the Wilcoxon’s test

* *p* < 0.05, ** *p* < 0.01, *** *p* < 0.001

Significant differences were also observed between the measurements of errors (χ^2^ (*N* = 30, *df* ^2^) = 6.42; *p* < 0.05). The BC group was found to score significantly lower than the HC group on the task measuring phonetic fluency (*Z* = −2.04; *p* < 0.05) as well as on the average cluster size in the same task (*Z* = −2.38; *p* < 0.05) at T3 (Table 3).

**Table 3:** Differences between the results before (T1), during (T2), and after chemotherapy (T3) in patients with cancer (BC) and healthy controls (HC), evaluated with Mann–Whitney *U* test

		BC (*n* = 30)			HC (*n* = 29)			BC (*n* = 30) v. HC (*n* = 29)		
		T1	T2	T3	T1	T2	T3	T1	T2	T3
		*M* (SD)	*M* (SD)	*M* (SD)	*M* (SD)	*M* (SD)	*M* (SD)	*Z*	*Z*	*Z*
Phonetic fluency	Word count	43.73 (10.1)	47.1 (10.09)	47.1 (12.62)	46.69 (12.96)	48.45 (11.9)	50.52 (12.85)	−1.08	−0.13	−1.15
	Error count	1.13 (2.29)	0.4 (1.1)	0.93 (1.31)	0.62 (0.98)	0.86 (2.36)	0.72 (1.25)	0.40	−0.70	0.61
	Cluster count	5.73 (3.12)	6.1 (2.98)	6.17 (2.74)	6.55 (2.29)	6.93 (2.99)	7.66 (2.35)	−1.67	−1.16	−2.04*
	Mean cluster size	3.58 (1.6)	3.67 (0.86)	3.28 (0.96)	3.96 (0.97)	4.15 (1.8)	4.12 (1.4)	−1.74	−0.57	−2.38*
	Switch count	33.17 (9.39)	36.5 (8.68)	37.07 (11.96)	34.86 (10.72)	36.48 (11.12)	37.03 (11.88)	−0.73	−0.13	0.08
Semantic fluency	Word count	51.63 (10.25)	52.13 (10.99)	52.57 (11.24)	54.21 (9.45)	53.62 (9.05)	57.31 (10.94)	−1.16	−0.61	−1.73
	Error count	0.73 (1.31)	0.8 (1.88)	1.17 (1.29)	0.38 (0.9)	0.83 (1.75)	0.62 (1.01)	1.07	−0.15	1.58
	Cluster count	13.83 (3.14)	13.67 (3.0)	14.57 (3.63)	14.79 (3.18)	13.86 (2.34)	15.28 (2.68)	−1.38	−0.12	−0.92
	Mean cluster size	8.01 (8.11)	7.12 (1.5)	6.19 (1.31)	6.26 (1.51)	6.51 (1.46)	6.52 (1.64)	0.76	1.44	−0.67
	Switch count	19.4 (4.88)	18.17 (4.22)	20.77 (3.69)	21.28 (4.12)	20.41 (4.07)	21.52 (6.11)	−1.73	−2.05*	−0.45

T1 – examination before, T2 – during, and T3 – after chemotherapy; *M* – mean; *SD* – standard deviation; *Z* – empirical value of the Mann–Whitney U test

* *p* < 0.05, ** *p* < 0.01, *** *p* < 0.001

The average cluster size was significantly smaller at T3 compared to T2 (*Z* = 2.01; *p* < 0.05) in the BC group (Table 2).

The number of phonetic switches in the BC group was significantly higher at T2 compared to T1 (*Z* = 2.62; *p* < 0.01) and at T3 compared to T1 (*Z* = 2.50; *p* < 0.01) (Table 2).

### Semantic fluency

It was shown that the HC group produced significantly more words in the task measuring semantic fluency at T3 than at T1 (*Z* = 2.62; *p* < 0.01) and at T3 compared to T2 (*Z* = 2.89; *p* < 0.01) in the HC group (Table 3).

A significant increase was also demonstrated in the number of semantic clusters at T3 compared to T2 (*Z* = 2.43; *p* < 0.05) in the HC group (Table 3).

In the BC group, the average number of clusters was significantly smaller at T3 compared to T2 in the semantic fluency task (*Z* = −2.85; *p* < 0.05).In this group, the number of semantic switches in the semantic fluency task was also significantly higher at T3 than at T2 (*Z* = 3.05; *p* < 0.01) (Table 2). Moreover, it was demonstrated that the BC group scored significantly lower than the HC group on the number of semantic switches in the semantic fluency task at T2 (*Z* = −2.05; *p* < 0.05) (Table 3).

## Discussion

The aim of this study has been to assess the relationship between phonetic and semantic verbal fluency and chemotherapy with AC scheme in women with breast cancer. To the best of our knowledge, the presented study is the first one in which the analysis took into account not only the number of produced words and the number of errors but also such qualitative parameters as the number and size of word clusters (mean cluster size), as well as the transitions between them (switches). An in-depth evaluation of a verbal fluency test enables the assessment of verbal functions and to draw conclusions regarding the speed of processing information, semantic memory, or the efficiency of executive functions [6, 7].

The results of the study showed an increase in the number of generated words under the phonetic criterion during and after chemotherapy in the group of women with breast cancer and at the third measurement in the group of healthy women. The improved performance in the test in both groups is most likely related to the learning effect. This effect occurs when measurements of cognitive functions are repeated using the same neuropsychological tests [31, 32]. The significantly smaller number of words before the start of chemotherapy compared to the examination during the treatment, observed only in the group of women with breast cancer, may indicate a relationship between verbal fluency to other factors, especially stress or anxiety associated with the first administration of chemotherapeutic treatment. The obtained results differ from the majority of studies published so far, which have not demonstrated any changes in the phonetic verbal fluency before and after chemotherapy [24, 25, 27, 28]. The differences are most likely not related to using the same set of letters, since the majority of researchers did the same [24, 25, 27], and only Wefel et al. [28] used alternate forms of verbal fluency test. The lack of learning effect in the adduced studies may result from the longer time between the pre- and post-chemotherapy examinations, associated with different schemes of chemotherapeutic treatment, such as cyclophosphamide, methotrexate, and fluorouracil (CMF), cyclophosphamide, epirubicin, fluorouracil (CEF), as well as from introducing treatments containing 5-fluorouracil (5-FU). The negative impact of 5-FU on cognitive function is linked to biochemical and structural changes in the brain, including the hippocampus area, confirmed by animal studies [33].

In the case of semantic fluency, an increase in the number of generated words was shown in the group of healthy women at the second and third measurements, whereas no similar relationship was observed in the group of women with breast cancer. It can thus be assumed that the group of healthy controls displayed the learning effect, which was not found in the group of women with breast cancer undergoing chemotherapy. Researchers [34, 35] suggest that the lack of improvement in test performance in spite of repeating the test procedure may be indicative of an impaired learning process. Cheng et al. [30] demonstrated even that women with breast cancer who received chemotherapy produced less words matching the specified semantic category (animal names) than healthy women.

In a study carried out on a group of healthy women, the learning effect was observed not only in the number of produced words which matched the phonetic and semantic criteria but also in the number of word clusters created. Each subsequent exposition to the test increased the knowledge of material and test procedure, which enabled a faster updating of semantic memory content. This in turn allowed them to make use of another group of words, linked phonetically or semantically. In the case of patients with breast cancer, the increase in the number of phonetic and semantic clusters was not observed; what was found instead was a decrease in the size of phonetic and semantic clusters post-chemotherapy, and an increase in the number of switches between clusters. The decreased size of produced word clusters seems to be indicative of an impaired access to semantic memory storage.

Impaired verbal memory retrieval after the administration of chemotherapy was also noted in the research of Quesnel et al. [26]. Likewise, Weis, Poppelreuter and Bartsch [36] pointed out that semantic memory and sustained attention are the basic cognitive functions which become impaired after the administration of chemotherapy. Results of animals study showed that doxorubicin generated imbalance in oxidative stress and antioxidant enzymes in brain damaging the structure of hippocampal area [37]. More frequent switches may be the manifestations of a mechanism to compensate for the difficulties in searching for words within one category. Therefore, the increased number of transitions between categories is a method of coping with the task so that the performance in this task remains at a level close to the baseline (the number of words did not decrease). Switches responsible for the executive aspect of speech are associated with the activation of prefrontal areas [9].

The results of a longitudinal study carried out among women with breast cancer treated with chemotherapy [38], in which the brain activity was evaluated with fMRI while performing working memory tasks (associated with executive functions), showed increased activation of the prefrontal areas before chemotherapy and 1 year post-chemotherapy, while the level of performance was maintained. The activation of brain areas not usually involved in the performance of a certain cognitive task was confirmed in several studies conducted on women with breast cancer receiving chemotherapy [10, 13, 39, 40]. Kesler, Bennet, et al. [13]. and Kesler, Kent, et al. [10] assessed activation of the brain during a memory, verbal learning and executive function task. They demonstrated decreased activation in the prefrontal cortex during retention and increased activation of various scattered areas of the brain during recall. Whereas Silverman et al. [40] showed an increase in metabolic activity in the frontal cortex and cerebellum during a recall in short-term memory task in breast cancer female patients receiving chemotherapy in contrast to a group not treated with CHTH, in whom the activation of parietal and occipital cortex was observed during the performance of the same task. It may be assumed that the compensatory activation of additional areas of the brain that allows to maintain the cognitive function within the norm [39, 40].

Summarizing the results of the presented study, it is worth to point out that while chemotherapy does not decrease verbal fluency, it has a negative impact on semantic memory. It is consistent with the results of previously published studies. The results of the study also call attention to the likely impact of compensatory mechanisms—more frequent switches—which allows to perform in a cognitive task at a level comparable to the state before the treatment was introduced.

### Limitations of the conducted study

The conducted study is not without its limitations. The intentional selection of women treated with the same scheme of chemotherapy may also be a limitation because of which the results should be only applicable to the patients administered by selected cytostatics. We chose healthy women matched by age and education as a control group. It would be highly recommended add another control group, e.g., women with BC treated with different antineoplastic treatment. Moreover, the intentional use of the same set of letters and categories during each measurement may lead to a learning effect. Nevertheless, in some studies [24, 25, 27] the learning effect was not observed even though the same sets of letters were used. The final component which deserves attention is the time of the third measurement. To minimize the influence of other usually administered anti-neoplastic therapy methods (like hormone therapy and radiation therapy), it was decided to conduct it immediately after the chemotherapy ended. It would be necessary to perform one more measurement at a longer time interval since its end.
